# Localization Properties of a Quasiperiodic Ladder under Physical Gain and Loss: Tuning of Critical Points, Mixed-Phase Zone and Mobility Edge

**DOI:** 10.3390/ma15020597

**Published:** 2022-01-13

**Authors:** Souvik Roy, Santanu K. Maiti, Laura M. Pérez, Judith Helena Ojeda Silva, David Laroze

**Affiliations:** 1 Physics and Applied Mathematics Unit, Indian Statistical Institute, 203 Barrackpore Trunk Road, Kolkata 700 108, India; souvikroy138@gmail.com; 2 Departamento de Física, FACI, Universidad de Tarapacá, Casilla 7D, Arica 1000000, Chile; lperez@uta.cl; 3 Grupo de Física de Materiales, Universidad Pedagógica y Tecnológica de Colombia, Tunja 150003, Colombia; judith.ojeda@uptc.edu.co; 4 Laboratorio de Química Teórica y Computacional, Grupo de Investigación Química-Física Molecular y Modelamiento Computacional (QUIMOL), Facultad de Ciencias, Universidad Pedagógica y Tecnológica de Colombia, Tunja 150003, Colombia; 5 Instituto de Alta Investigación, CEDENNA, Universidad de Tarapacá, Casilla 7D, Arica 1000000, Chile; dlarozen@uta.cl

**Keywords:** localization phenomena, AAH ladder, physical gain and loss, inverse participation ratio, mobility edge, mixed phase

## Abstract

We explore the localization properties of a double-stranded ladder within a tight-binding framework where the site energies of different lattice sites are distributed in the cosine form following the Aubry–André–Harper (AAH) model. An imaginary site energy, which can be positive or negative, referred to as physical gain or loss, is included in each of these lattice sites which makes the system a non-Hermitian (NH) one. Depending on the distribution of imaginary site energies, we obtain balanced and imbalanced NH ladders of different types, and for all these cases, we critically investigate localization phenomena. Each ladder can be decoupled into two effective one-dimensional (1D) chains which exhibit two distinct critical points of transition from metallic to insulating (MI) phase. Because of the existence of two distinct critical points, a mixed-phase (MP) zone emerges which yields the possibility of getting a mobility edge (ME). The conducting behaviors of different energy eigenstates are investigated in terms of inverse participation ratio (IPR). The critical points and thus the MP window can be selectively controlled by tuning the strength of the imaginary site energies which brings a new insight into the localization aspect. A brief discussion on phase transition considering a multi-stranded ladder was also given as a general case, to make the present communication a self-contained one. Our theoretical analysis can be utilized to investigate the localization phenomena in different kinds of simple and complex quasicrystals in the presence of physical gain and/or loss.

## 1. Introduction

The phenomenon of Anderson localization [1,2] was explored almost seven decades ago and it remains a highly active area of research in the discipline of condensed matter physics [3,4] since the localization behavior helps us understand how the mobility of carriers is affected by disorder. After many years of theoretical prediction, experimental verification was performed using an optical lattice setup [5,6] and is nowadays being observed in many cases.

It is well known that for a 1D system with random (uncorrelated) site energies (diagonal disordered system), all energy eigenstates are exponentially localized regardless of the strength of the disorder [1,2]. This indicates that the critical disorder strength $W_{c}=0$ (*W* measures the disorder strength), and thus, such a system is quite trivial as we do not have any option to tune the localization behavior. The situation becomes very interesting when a correlation is imposed among the site energies. There exist many examples of correlated disordered systems that are widely used to study localization phenomena, and among them, the most notable system is probably the Aubry–André–Harper (AAH) [7,8,9,10,11,12,13,14,15,16,17] where site energies are correlated in the cosine form. Unlike in the 1D random disordered system, a 1D AAH chain provides a finite critical point of phase transition, described by the relation $W_{c}=2t$ (*t* being the nearest-neighbor hopping (NNH) strength) [8,18,19,20]. For $W<W_{c}$, all energy eigenstates are perfectly conducting, whereas beyond this critical limit these states are localized. Thus, a metal-to-insulator (MI) transition occurs at $W=W_{c}$. Though a finite critical point is obtained for a 1D AAH chain, one never encounters any mixture of both conducting and localized states in the energy band spectrum, i.e., a *mixed-phase* (MP) [18,20] does not appear. The existence of an MP zone is of course very crucial in many contexts, and it is the primary requirement to have a *mobility edge* (ME) which separates the conducting zone from the insulating one in the energy eigenspectrum [18,20,21,22,23,24].

**Figure 1:**
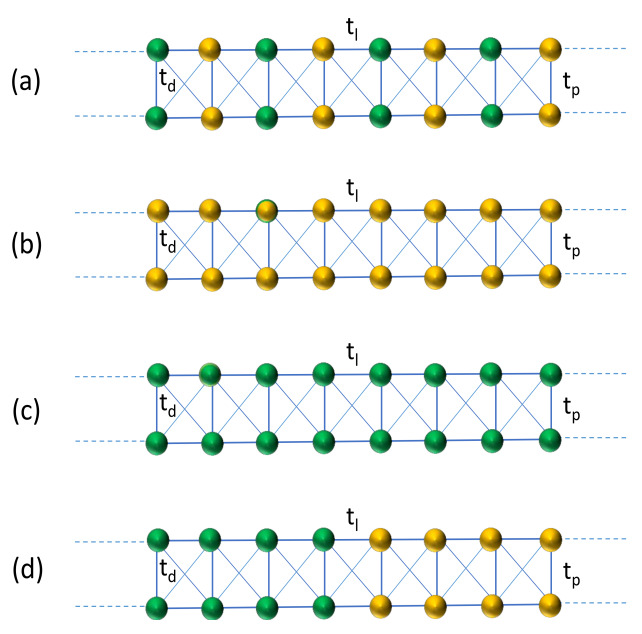
(Color online). Schematic diagrams of non-Hermitian Aubry–André–Harper ladders following the four different arrangements of the imaginary site energies those are respectively presented in (**a**), (**b**), (**c**) and (**d**). The green ball corresponds to the lattice site where negative imaginary energy (loss term) is added, while the orange ball indicates the site where positive imaginary energy is included (gain term). The parameters $t_{l}$ and $t_{p}$ refer to the intra- and inter-strand hopping integrals, respectively, and $t_{d}$ describes the diagonal hopping between the two strands.

To have the mixed-phase energy window and mobility edge, two basic requirements are (i) coupling at least two such 1D AAH chains to form a ladder-like geometry and (ii) diagonal coupling between the strands (see Figure 1). In this context, it is relevant to note that for a strictly 1D AAH chain, one can obtain the mixture of both conducting and insulating states provided the hopping of electrons is not limited to nearest-neighbor sites [25]. In that case, the critical disorder strength is no longer a constant; rather, it depends on the discrete energy eigenvalues. In our present work, we do not consider that situation, and it will maybe be taken into account in any of our forthcoming works, as our present focus is something different. For a two-stranded AAH ladder, a finite energy window is obtained for the mixed phase resulting in a mobility edge [18], and the primary aim of our work is to establish a prescription for tuning the MP window together with the ME.

To substantiate this fact, we included the effect of environmental interaction [26,27,28,29,30,31,32,33,34] in the system, which is actually unavoidable in many realistic situations. Phenomenologically, such an interaction is introduced by adding an imaginary site energy in a lattice site, which makes the system a non-Hermitian (NH) one [35,36,37,38,39,40]. Depending on the sign—which is positive or negative—of the imaginary site energy, we have physical gain or loss in the system. This physical gain/loss might be associated with energy or electrons or even bosonic particles as well, and has already been established in different contemporary works [41,42,43,44,45]. In our analysis, we add imaginary site energies at all distinct lattice sites, along with the cosine modulation following the AAH form, and studied the interplay between the environmental interaction (viz, the NH quantity) [46,47,48,49,50,51] and the correlated disorder on the localization phenomena. Both balanced and imbalanced NH distributions are taken into account [52,53] for a comprehensive analysis.

We simulate the two-stranded NH AAH ladder within a tight-binding (TB) framework, where both the strands are assumed to be identical in nature. Such a ladder can be decoupled into two effective 1D chains, and the localization phenomena of the parent ladder is investigated by critically analyzing the localization behavior of these individual 1D chains [18,20]. The interesting thing is that the two decoupled chains provide two distinct critical points of MI transition, and thus an MP zone appears. The existence of the MP window suggests the availability of an ME in the energy band spectrum. The conducting nature of each energy eigenstate is described by determining the inverse participation ration (IPR) which gives a very good measure as it is directly involved with the participation of particles at different lattice sites. For a conducting state, IPR becomes vanishingly small, whereas it goes to unity for the absolute localized state. Using IPRs of all the distinct energy states, we eventually computed average inverse participation ratio (AIPR) [20] which measures the conducting behavior of the system. AIPR →0 denotes the metallic phase [54,55], while for the insulating phase [56,57] AIPR →1.

The key features that we want to address in this work are as follows. (i) The specific role of environmental interaction [58,59,60,61,62,63,64,65,66] (NH factor), which is introduced through imaginary site energies, on critical point of phase transition. It essentially gives us a possible tuning mechanism of the critical disorder strength. (ii) The modification of mixed-phase [67,68] energy region and ME, by means of the NH quantity. (iii) Decoupling of the parent ladder system into two effective 1D NH AAH chains. A general case considering a multi-stranded system is briefly discussed at the end, as an extension, for the sake of completeness. These features have not been well explored in the literature, to the best of our knowledge. We believe that the present analysis may provide some insights into the localization phenomena in different kinds of correlated disordered systems subjected to physical gain/loss, and certainly bring a new route for designing more controlled devices.

Following the above brief introduction (Section 1), the remaining parts of the paper are arranged as follows. In Section 2, we describe the NH AAH ladder and the distribution of physical gain and loss. Subsequently, we present the TB Hamiltonian, decoupling the prescription of the ladder and the methodology for studying the localization behavior. In Section 3, we present all the essential results and thoroughly discuss them one by one. The specific role of the NH factor is critically discussed. Finally, in Section 4, we summarize our key findings.

## 2. Non-Hermitian AAH Ladder, Decoupling Procedure and the Theoretical Framework

### 2.1. Two-Stranded NH AAH Ladder and the Tight-Binding Hamiltonian

Let us start with Figure 1, where the schematic diagrams of two-stranded ladders are shown. In each case, two identical strands are vertically stacked to form a ladder, where the site energies are modulated by the cosine form obeying the well-known AAH model. The two colored balls are used to refer two kinds of imaginary site energies that are added into the parent AAH lattices, i.e., the base site energies are described in the AAH form where the imaginary part is added, to make the system a non-Hermitian one. In one type of site, a positive imaginary quantity is added (orange site), described as ‘iδ’ where δ measures the strength of the NH factor. This is referred as ‘physical gain’ in the system, which can be the gain of energy or we can also call it as the gain of particles. In the other type of site, represented by green ball, a negative energy is included in the form ‘−iδ’. This is known as ‘physical loss’ in the system. Both the gain and loss in the system are involved with the interaction of the physical system with the environment.

We can arrange the green and orange balls, or more precisely, we can say that the gain and loss terms can be added into the system, in many ways, but here, we consider only four distinct configurations and they are sequentially represented in Figure 1. When the green and orange balls are alternately arranged, we obtain the most stable configuration, and it is called the balanced type-I configuration (see Figure 1a). The gain/loss configurations used in Figure 1b,c are referred as imbalanced type-I and imbalanced type-II, respectively. In one case, all sites are subjected to a constant gain term, while for the other ladder a loss factor is added into the lattice sites. Finally, the ladder shown in Figure 1d is called balanced type-II, where in one half, the gain term is added and the other half loss factor is included. For all these four types of NH AAH ladders, we critically investigate the localization properties and specific role of the NH factor.

Any such ladder can be simulated within a tight-binding framework, and the general form of the TB Hamiltonian looks like:

(1) $$\begin{array}{cc} H & =\sum_{n,m}\epsilon_{n,m}^{eff}\;c_{n,m}^{†}c_{n,m}+\sum_{n,m}t_{l}\left(c_{n+1,m}^{†}c_{n,m}+h.c.\right)\\ \; & +\sum_{n,m}t_{p}\left(c_{n,m+1}^{†}c_{n,m}+h.c.\right)+\sum_{n,m}t_{d}[(c_{n+1,m+1}^{†}\\ \; & c_{n,m}+c_{n+1,m}^{†}c_{n,m+1})+h.c.]. \end{array}$$

The integer *n* is used to describe the lattice sites in each strand which runs from 1 to *N* (*N* being the total number of lattice sites in each single strand), whereas the strand index is described by the integer *m* and it runs from 1 to *M* (*M* denotes the total number of strands). $c_{n,m}^{†}$, $c_{n,m}$ are the conventional Fermionic operators at the lattice site (n,m) (*n*th site of *m*th strand). The parameters $t_{l}$, $t_{p}$ and $t_{d}$ describe the intra-strand, inter-strand and diagonal hopping integrals, respectively, as clearly depicted in Figure 1. $\epsilon_{n,m}^{eff}$ is the effective site energy which is the sum of two terms: one is associated with the cosine modulation, i.e., the AAH term and the other is the imaginary site energy in the form of +iδ or −iδ. Since the AAH site energies are taken to be identical for both strands, we can express $\epsilon_{n,m}^{eff}$ as

(2) $$\epsilon_{n,m}^{eff}=\epsilon_{n}^{eff}=\epsilon_{n}+\delta_{n}$$

where:

(3) $$\epsilon_{n}=W\cos\left(2\pi bn\right).$$

The index *m* in effective site energy is removed without any loss of generality, as for all the strands, $\epsilon_{n}$s have an identical form. *W* measures the AAH modulation strength, which is usually referred to as the correlated disorder strength, and *b* is an incommensurate quantity. In our analysis, we chose $b=(1+\sqrt{5})/2$ (golden mean) [13,69,70]. This typical value is widely used in the literature, though any other irrational number can also be considered, and the physics will remain unchanged. $\delta_{n}$ represents the gain or loss term, viz, $\delta_{n}=i\delta$ or $\delta_{n}=-i\delta$ depending on the arrangement of the orange and/or green colored balls in the AAH ladders (see Figure 1).

### 2.2. Decoupling of Two-Stranded AAH Ladder into Two Effective 1D Chains

The decoupling of the ladder into individual 1D chains is an important perspective for analyzing the mixed-phase window, mobility edge phenomenon, and the overall localization behavior of a full system. The decoupling can be made in several ways, as already noted by different groups including us [18,19,20]. Here, we use a simple prescription that is described as follows.

Starting from the Schrödinger equation H|Ψ〉=E|Ψ〉, we can write the tight-binding difference equation as

(4) $$\begin{array}{cc} \left(E-\epsilon_{n}^{eff}\right)\Psi_{n,m} & =t_{l}\left(\Psi_{n+1,m}+\Psi_{n-1,m}\right)+t_{p}(\Psi_{n,m+1}\\ \; & +\Psi_{n,m-1})+t_{d}(\Psi_{n+1,m+1}+\Psi_{n-1,m+1}\\ \; & +\Psi_{n+1,m-1}+\Psi_{n-1,m-1}) \end{array}$$

where $\Psi_{n,m}$’s are the wave amplitudes. As the site energies are identical along each rung (referred as the *y* direction), we can write a plane wave solution along the *y* direction, and hence:

(5) $$\Psi_{n,m}=B\;\psi_{n}^{q}\;e^{imk_{y}a}$$

where *B* is a constant factor, $\psi_{n}^{q}$ is the wave amplitude for the *x* direction, $k_{y}$ is the wave vector, *q* is the strand index and *a* is the lattice spacing. Substituting such wave amplitude in the above equation, we have:

(6) $$\begin{array}{cc} \left(E-\epsilon_{n}^{eff}\right)B\;\psi_{n}^{q}\;e^{imk_{y}a} & =t_{l}\left(B\;\psi_{n+1}^{q}\;e^{imk_{y}a}+B\;\psi_{n-1}^{q}\;e^{imk_{y}a}\right)+t_{p}(B\;\psi_{n}^{q}\;e^{i\left(m+1\right)k_{y}a}\\ \; & +B\;\psi_{n}^{q}\;e^{i\left(m-1\right)k_{y}a})+t_{d}(B\;\psi_{n+1}^{q}\;e^{i\left(m+1\right)k_{y}a}+B\;\psi_{n-1}^{q}\;e^{i\left(m+1\right)k_{y}a}\\ \; & +B\;\psi_{n+1}^{q}\;e^{i\left(m-1\right)k_{y}a}+B\;\psi_{n-1}^{q}\;e^{i\left(m-1\right)k_{y}a}). \end{array}$$

Now, eliminating the term $B\;e^{imk_{y}a}$ from both sides of the above equation, we obtain:

(7) $$\begin{array}{cc} \left(E-\epsilon_{n}^{eff}\right)\psi_{n}^{q}\; & =t_{l}\left(\psi_{n+1}^{q}+\psi_{n-1}^{q}\right)+t_{p}\left(\psi_{n}^{q}\;e^{ik_{y}a}+\psi_{n}^{q}\;e^{-ik_{y}a}\right)+t_{d}(\psi_{n+1}^{q}\;e^{ik_{y}a}\\ \; & +\psi_{n-1}^{q}\;e^{ik_{y}a}+\psi_{n+1}^{q}\;e^{-ik_{y}a}+\psi_{n-1}^{q}\;e^{-ik_{y}a}). \end{array}$$

Doing some simple algebraic steps, we reach the expression:

(8) $$\begin{array}{cc} \left(E-(\epsilon_{n}^{eff}+2t_{p}\cos\left(k_{y}a\right)\right)\psi_{n}^{q}\; & =\left(t_{l}+2t_{d}\cos\left(k_{y}a\right)\right)(\psi_{n+1}^{q}+\psi_{n-1}^{q}). \end{array}$$

The wave vector $k_{y}$ is governed by the relation $k_{y}a=q\pi /\left(M+1\right)$. Now, considering M=2 (q=1, 2), we eventually reach the following equations:

(9a) $$\begin{array}{cc} \left[E-\left\{W\cos\left(2\pi bn\right)+\delta_{n}+t_{p}\right\}\right]\psi_{n}^{1} & =\;\left(t_{l}+t_{d}\right)\left[\psi_{n+1}^{1}+\psi_{n-1}^{1}\right], \end{array}$$

(9b) $$\begin{array}{cc} \left[E-\{W\cos\left(2\pi bn\right)+\delta_{n}-t_{p}\right\}]\psi_{n}^{2} & =\;\left(t_{l}-t_{d}\right)\left[\psi_{n+1}^{2}+\psi_{n-1}^{2}\right]. \end{array}$$

These equations can be further expressed in compact forms as

(10a) $$\begin{array}{cc} \left[E-\epsilon_{n}^{1,eff}\right]\psi_{n}^{1} & =t^{1,eff}\left[\psi_{n+1}^{1}+\psi_{n-1}^{1}\right] \end{array}$$

(10b) $$\begin{array}{cc} \left[E-\epsilon_{n}^{2,eff}\right]\psi_{n}^{2} & =t^{2,eff}\left[\psi_{n+1}^{2}+\psi_{n-1}^{2}\right] \end{array}$$

The effective site energies are:

$$\epsilon_{n}^{1,eff}=W\cos\left(2\pi bn\right)+\delta_{n}+t_{p}$$

and:

$$\epsilon_{n}^{2,eff}=W\cos\left(2\pi bn\right)+\delta_{n}-t_{p},$$

and the effective hopping integrals are: $t^{1,eff}=\left(t_{l}+t_{d}\right)$ and $t^{2,eff}=\left(t_{l}-t_{d}\right)$. Equations (10a) and (10b) represent two effective 1D chains which are obtained by decoupling the parent NH AAH ladder. Now, depending on the distribution of the gain/loss term ($\delta_{n}$), we obtain different effective site energies for the decoupled chains.

### 2.3. Theoretical Prescription

We compute the localization behavior of each energy eigenstate by studying the inverse participation ratio. For any normalized eigenstate (say) $|\phi_{q}\rangle =\sum_{n=1}^{N}|\Psi_{n}^{q}\rangle$, the IPR is defined as

(11) $$IPR_{q}=\sum_{n=1}^{N}{\left|\Psi_{n}^{q}\right|}^{4}.$$

This quantity clearly describes whether a state is conducting or non-conducting in behavior. For the conducting state, we obtain IPR${}_{q}\to 0$, while it becomes to unity for the non-conducting sate. It is usually quite difficult to have IPR${}_{q}\to 1$ when *N* is finite, since only in the asymptotic limit (N→∞) can we attain the ‘absolute localized phase’.

Determining all the IPR${}_{q}$s for the individual energy eigenstates, we finally compute the average inverse participation ratio that describes the conducting properties of the system. The AIPR [20,54,55,56,57] is defined as

(12) $$AIPR=\langle IPR\rangle =\frac{1}{N}\sum_{q=1}^{N}IPR_{q}.$$

For the conducting phase, AIPR →0, while AIPR →1 denotes the localized phase of the system. For the intermediate values of AIPR, we obtain the mixed phase.

## 3. Numerical Results and Discussion

In what follows, we present and discuss our results which include the energy band spectra, the control of the critical point of transition from metallic to insulating phase, the regulation of mixed-phase window and related issues. From now on, we abbreviate ‘balanced type’ as BT and ‘imbalanced type’ as IT, for simplification, and here we thoroughly investigate the results of four different types of NH AAH ladders (BT-I, IB-I, IB-II and BT-II), those are schematically shown in Figure 1.

Before presenting the results, let us mention the parameter values which are kept constant throughout the computation. We set $t_{l}=1.5\;$eV, $t_{p}=1\;$eV and $t_{d}=0.5\;$eV. The number of lattice sites in each strand of the ladder is fixed at N=250, unless specified. The other parameters that are not so common are mentioned in the appropriate places. All the energies are measured in unit of electron-volt (eV).

### 3.1. Two-Stranded AAH Ladders in Presence of Physical Gain and/or Loss

The central focus of our work was to investigate the critical role of physical gain and/or loss on electronic localization. Before coming to that part, it is indeed necessary to analyze the energy level diagram, as this is the most fundamental thing to understand a physical system. From our mathematical analysis, we find that a two-stranded ladder is decoupled into two effective 1D NH AAH chains, described by the relations given in Equations (10a) and (10b). Both these two decoupled chains obtain effective site energies and NNH integrals. The chain possessing the higher effective hopping integral (viz, $t_{l}+t_{d}$) is called chain-1, while the other chain with the lower hopping strength (i.e., $t_{l}-t_{d}$) is called chain-2.

#### 3.1.1. Eigenvalue Spectrum

**Figure 2:**
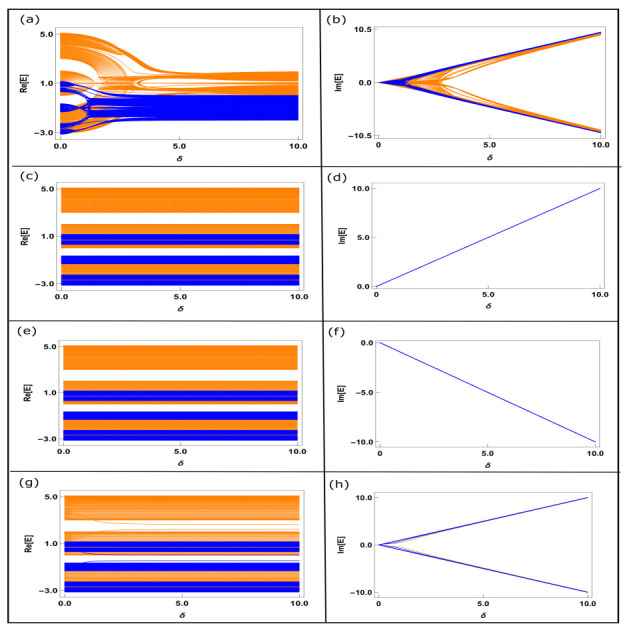
(Color online). Variation of the real (first column (**a**,**c**,**e**,**g**)) and imaginary (second column (**b**,**d**,**f**,**h**)) eigenvalues with the non-Hermitian parameter δ for the four different ladders, where the 1st, 2nd, 3rd and 4th rows correspond to the BT-I, IT-I, IT-II and BT-II cases, respectively. Here, we fix the strength of the Aubry–André–Harper site potential W=1 in each case. The orange and blue colors are associated with the decoupled chain-1 and chain-2, governed by the relations Equations (10a) and (10b), respectively.

In the presence of the NH factor, the eigenvalues become complex, and in order to have a clearer picture, in Figure 2, we show the variations of both the real and imaginary parts of the eigenvalues as a function of δ, and for all the four different types of ladders. The orange and blue colors are involved with the chain-1 and chain-2, respectively. Several interesting features emerge from the band spectra. For the BT-I case, a significant change in real eigenvalues with δ was obtained when δ is quite small (Figure 2a), while the effect becomes relatively weaker for higher δ. This is primarily due to the interplay between AAH potential and the NH quantity. For low δ, both these factors contribute and the effect of AAH modulation becomes gradually diminished with increasing δ. Because of this fact, we initially obtain three sub-bands in the real eigenvalue spectrum which is the generic feature of AAH modulation, while this behavior almost vanishes when δ is reasonably large. Instead of three sub-bands, we almost obtain a continuous band. As alternate sites contain gain and loss terms for this BT-I case, the real eigenvalues are practically constant beyond some critical limit of δ. The finite separation between the orange and blue bands are associated with the effective tight-binding parameters of the decoupled chains.

For the other two ladders denoted by IT-I and IT-II, the real eigenvalues are insensitive to δ (as can be seen in Figure 2c,e), which is easy to understand since all the sites contain a constant gain or loss term. Finally, for the ladder where the gain term is included in one half and the loss term is added in the other half (BT-II ladder), a slight variation in the real spectrum is obtained, and that is also for the smaller δ (Figure 2g).

The variations of imaginary eigenvalues with δ are also quite interesting. For the IT-I and IT-II ladders, the eigenvalues of the decoupled chains (chain-1 and chain-2) completely merge with each other (Figure 2d,f), and the increment or reduction in eigenvalues with δ is very simple to understand since all sites contain +iδ and −iδ, respectively, for the IT-I and IT-II ladders, respectively. Thus, the variation follows a straight line with δ. Similarly to the real eigenvalue spectrum, the imaginary eigenvalues are also highly sensitive to the NH factor for the BT-I ladder, and this effect is more prominent for a smaller δ (Figure 2b). Furthermore, finally, for the BT-II ladder, the imaginary eigenvalues follow almost linear dependence both along the positive and negative directions (Figure 2h). This is solely associated with the two halves of the ladder that contain positive and negative imaginary site energies.

Thus, what we find from the real and imaginary eigenvalue spectra shown in Figure 2 is that for the AAH ladder with balanced gain and loss distribution, the eigenvalues are most sensitive and a more complex pattern is obtained when δ is relatively small, i.e., comparable to the AAH modulation strength. This is due to the combined effects of these two factors (viz, *W* and δ).

#### 3.1.2. Tuning of Critical Points, Mixed-Phase Zone and Mobility Edge

Now, we focus on the central feature of our analysis, i.e., the tuning of critical points and the localization phenomena by means of the NH parameter δ.

**Figure 3:**
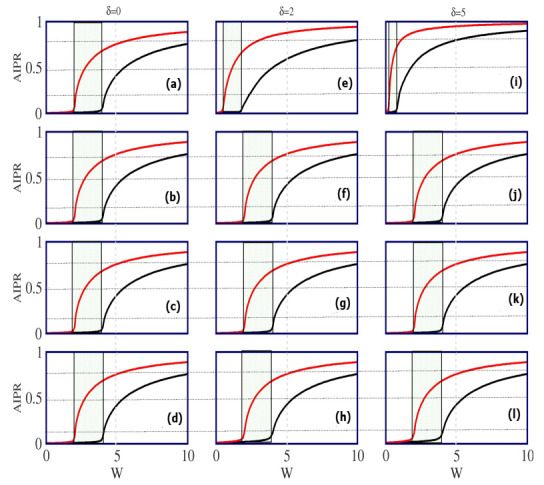
(Color online). Dependence of the average inverse participation ratio with the Aubry–André–Harper modulation strength *W*, where in each spectrum, the black and red lines are associated with chain-1 and chain-2, respectively. The results are shown (**a**–**l**) for three typical δ values (placed in the three different rows), where the four different rows (top to bottom) correspond to the BT-I, IT-I, IT-II and BT-II ladders, respectively. The light-shaded region denotes the mixed-phase window, that is tuned by means of δ.

In Figure 3, we show the dependence of AIPR as a function of the correlated disorder strength *W* for all four AAH ladders, at three distinct values of δ. In each spectrum, the AIPR-δ curves associated with chain-1 (black line) and chain-2 (red line) are superimposed. It is observed that the two chains exhibit two separate critical points of transition from the metallic phase to the insulating one. Here, we are not expecting a sharp jump of AIPR from its almost zero value to unity, since the system size is finite, as mentioned previously. However, the signature of the phase transition is clearly reflected. Because of the two different critical points, we obtain a window of *W* for which all the eigenstates of one chain (chain-1) are completely extended in behavior, while for the other chain (chain-2), all the states are localized. As a result of this, a mixed-phase window appears (lightly shaded region), and at the same time, we obtain a mobility edge that creates a partition among the conducting and insulating states. When δ=0, the critical disorder strengths of the transitions of the two decoupled chains are: $W_{c1}=2\left(t_{l}+t_{d}\right)$ and $W_{c2}=2\left(t_{l}-t_{d}\right)$ (two times the effective NNH strength for the diagonal AAH chain). This is well known in the literature, and has been discussed by many groups including ourselves [18,19,20]. The main goal of this communication was to verify whether the transition points can be tuned by means of environmental interaction δ, in the presence of the cosine modulation. If yes, then it will definitely be an interesting result and might be utilized in several ways, especially in the context of localization phenomena.

Carefully looking into the spectra given in the second and third rows of Figure 3, we can see that, for the IT-I and IT-II ladders, there is absolutely *no* change in the transition points, and thus the MP window, due to δ. A very slight change can be noticed for the BT-II ladder (last row), and an appreciable modification is only obtained for the BT-I AAH ladder (top row). These features can be explained as follows. In the presence of the physical gain and/or loss, a phase factor is introduced at each and every lattice site. Depending on the gain or loss term, the probability of finding a particle at different lattice sites becomes increased or decreased. When all sites possess either a gain or loss factor, for instance, in the IT-I and IT-II ladders, the phases are the same at all the sites which essentially do not produce any new scattering and hence there is no effect on the localization. In the BT-II ladder, a phase mismatch only occurs at one particular place (the center of the ladder), where the sign reversal of δ takes place. Since at all other regions the phases are identical, the scattering of particles only at the center does not produce any appreciable effect, and it becomes much weaker with the increasing ladder length. The noticeable effect of δ is only observed in BT-I ladder as in this case, a regular phase change takes place in every alternate lattice sites, and thus the scattering becomes stronger which yields the localization. Naturally, the critical points of phase transition for both the two decoupled chains, i.e., $W_{c1}$ and $W_{c2}$ become reduced with the increase in the interaction parameter δ, as it enhances more scattering at the lattice sites.

The results presented in Figure 3 clearly indicate that the BT-I ladder is most sensitive on the interaction parameter δ. Therefore, in the rest of our analysis, we only concentrate on the BT-I ladder.

**Figure 4:**
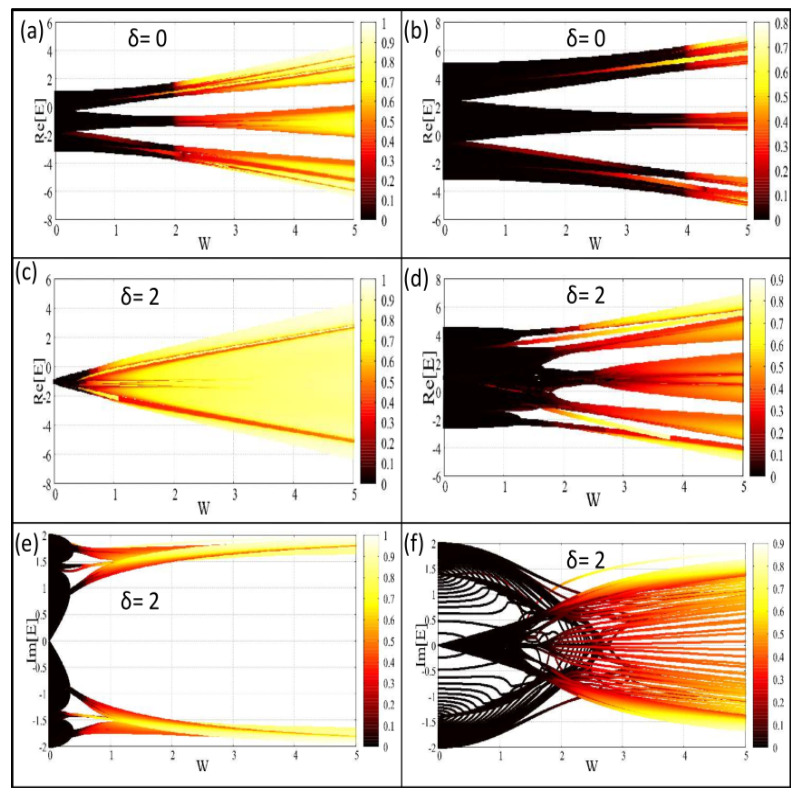
(Color online). Energy resolved inverse participation ratio. Density plot of inverse participation ratios of different eigenstates as functions of the Aubry–André–Harper modulation strength *W* and the eigenvalues for the BT-I ladder, where the first (**a**,**c**,**e**) and second columns (**b**,**d**,**f**) are associated with the chain-2 and chain-1, respectively. For δ=0, we only have real eigenvalues, whereas for non-zero δ, we have both real and imaginary parts, and we plot them into two separate rows (second and third).

In order to see the direct effect of δ on different eigenstates and for a better viewing of the mobility edge, in Figure 4, we show the density plot of IPRs of all the eigenstates as functions of the eigenvalues and the AAH modulation strength. The two columns are associated with the two decoupled chains of the BT-1 ladder. The results for δ=0 are also included (where all the eigenvalues are real in nature), to obtain a comparative analysis on IPRs when δ is finite. For δ=0, we see that in a wide range of *W*, all the eigenstates of chain-1 exhibit conducting behavior (see Figure 4b), while for the other chain (chain-2), all the states become localized at a low *W* (Figure 4a). This reveals the co-existence of the conducting and localized eigenstate, and thus the mobility edge. The appearance of the mixed phase is fully consistent with the results discussed in Figure 3 for the BT-I ladder. For non-zero δ, the density plots are quite anomalous, especially for the imaginary case eigenvalues, though the band splitting and gapped-like spectrum still persists. These are the generic features of AAH systems. What we clearly find is that states become localized at a much lesser *W* when we include the effect of δ which clearly corroborates our findings in Figure 3 for the BT-I ladder. The physical explanation behind the reduction in critical points of both the two decoupled chains with δ was already given earlier.

**Figure 5:**
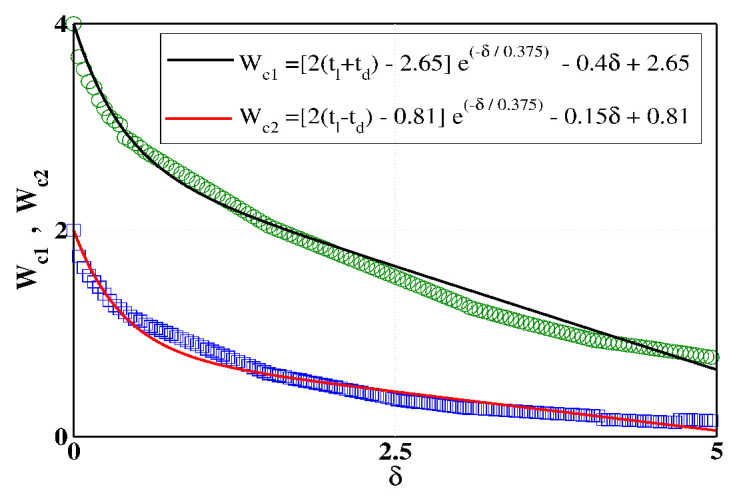
(Color online). Dependence of two critical points $W_{c1}$ and $W_{c2}$ on the interaction parameter δ for the BT-I ladder. The green circles and blue squares, associated with the chain-1 and chain-2, are numerically computed, and these data points are fitted with appropriate mathematical expressions which are given in the inset. The two expressions give a very good fit of the numerical data.

Based on the characteristic features of Figure 3 and Figure 4, it is indeed necessary to check how the critical points are affected by a continuous variation of δ. The results are presented in Figure 5, where the variations of $W_{c1}$ and $W_{c2}$ are shown as a function of δ for the BT-I ladder. We consider a large range of δ, and compute the critical points of transitions for the two decoupled chains. For both these chains, the critical disorder strengths varies continuously, providing a smooth variation with respect to δ. The green circles and the blue squares represent the numerically obtained data points, and these data are fitted well with closed mathematical forms in terms of δ. Here, it is relevant to note that there is *no* physical reason for getting this dependence of the critical point with δ. It is always easier to understand any property if we have a closed form or mathematical expression in terms of the variable parameter(s) associated with the system. We can therefore even estimate the value of the critical point even for any arbitrary values of δ. For our case, we find that the data points $W_{c1}$ and $W_{c2}$ are fitted well with these functional forms. One can of course fit the data with other functional form(s), but we need to focus on the best fit, and here we confirm these relations through numerous attempts. The results of Figure 5 reveal that the difference between the two curves is initially large, but the difference gradually decreases with the increasing δ. The gap practically disappears for a sufficiently large δ (not shown here in the figure), which we confirm through our detailed numerics.

This is quite easily understandable, as for a much higher δ, both the two chains become localized for a vanishingly small *W*. Due to the reduction in critical disorder strengths with δ, the width of the mixed-phase window becomes narrowed providing a shifting of the mobility edge. Eventually, at the stage when the width becomes small to the point of vanishing, it is difficult to achieve a mobility edge since the coexistence of both the localized and extended states is the primary requirement to have the mobility edge.

**Figure 6:**
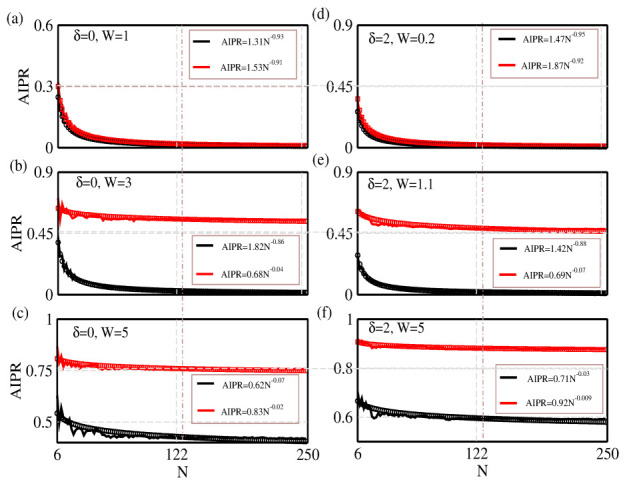
(Color online). Scaling behavior. Variation of the average inverse participation ratio on the number of lattice sites *N*, both in the absence (left column (**a**–**c**)) and presence (right column (**d**–**f**)) of the interaction parameter δ for the BT-I ladder. The red color is associated with chain-2, while for the other chain, the black color is used, similarly to in Figure 3. We selectively choose *W* such that we can only obtain the conducting phase, or the mixed phase or the fully localized one. These three different cases are presented in the top, middle and bottom rows respectively. In each case, we provide a closed-form analytical relation that fits very well with the numerically extracted data.

To make the present analysis a self-contained one, here we focus on the scaling behavior of AIPR on the system size *N*. The results are presented in Figure 6 for the BT-I ladder under different input conditions of δ and *W*. Very nicely, we find that for each and every set of input parameters, AIPR follows a nice pattern with *N*, and therefore, we can fit the data using a simple closed form (given in the inset of each sub-figure).

### 3.2. *M*-Stranded Ladder: A General Case

To make the present analysis a self-contained one, we finally focus on an *M*-stranded ladder. The value of *M* can be anything. Any such ladder is constructed by vertically stacking *M* number of identical AAH NH chains, and the neighboring chains are coupled in the same way as was discussed above for the two-stranded ladder (viz, vertically as well as diagonally). As the site energies in each rung, possessing *M* number of lattice sites, are identical, we can write a plane wave solution along the *y* direction, and following the same mathematical prescription given in Section 2.2, we can decouple *M*-stranded ladder into *M*-distinct effective 1D chains. The different equation of *M* such chains involving the wave amplitudes are written in a compact form as

(13) $$\begin{array}{cc} \left[E-\left\{W\cos\left(2\pi bn\right)+\delta_{n}+2t_{p}\cos\left(\frac{q\pi }{M+1}\right)\right\}\right]\psi_{n}^{q} & \\ =\left\{t_{l}+2t_{d}\cos\left(\frac{q\pi }{M+1}\right)\right\}\left[\psi_{n+1}^{q}+\psi_{n-1}^{q}\right] \end{array}$$

where the integer *q* runs from 1 to *M*. Once we obtain the decoupled chains, we can easily determine the critical disorder strengths $W_{cm}$ for the *M* different effective chains by computing AIPR as a function of the AAH modulation strength *W*.

**Figure 7:**
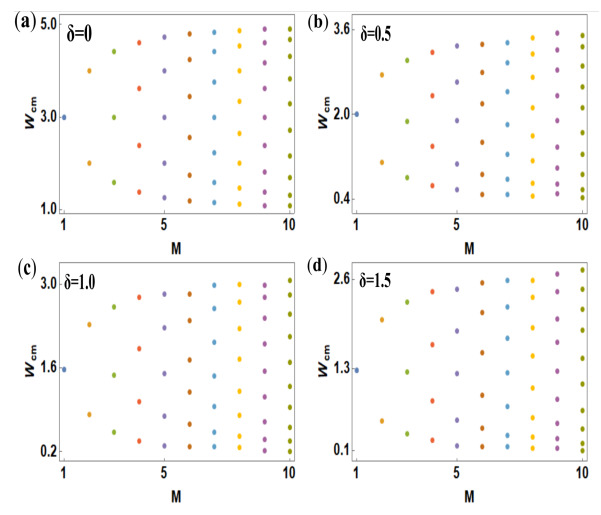
(Color online). Dependence of critical points $W_{cm}$ of phase transition on the non-Hermitian parameter δ for different stranded Aubry–André–Harper ladders. We compute the results for the BT-I case only by varying the number of strands M=1 to M=10. The colored dot points represent the critical disorder strengths, which follow quite a regular pattern. Four different δ values are taken into account, as typical examples, and the results are presented in (**a**), (**b**), (**c**) and (**d**).

In Figure 7, we plot the critical points of transition for different stranded AAH ladders at four typical values of the NH parameter δ. The results are computed by varying *M* from 1 to 10, where we only choose the BT-I case since this type of ladder is most sensitive to δ. The values of the critical disorder strengths are represented by the colored dot points in each spectrum. At a first glance, we see that the dot points follow an almost regular pattern, but a careful inspection reveals that the uniformity starts to become distorted with the increasing strength of δ. Most importantly, we find that the values of $W_{cm}$ gradually decrease with δ. The reduction in critical points is solely associated with more scattering of particles in the regime of higher δ. The key feature that emerges from the spectra is that, with the increasing number of strands, more and more distinct critical points are generated which yield multiple mixed-phase zones in the energy spectrum. Because of this, we have a finite possibility of obtaining multiple mobility edges. Moreover, we can tune the mixed-phase windows as well as the mobility edges by means of the interaction parameter δ. These aspects are highly significant to achieve high to low conducting switching action and vice versa, and to selectively tune the localization properties with the help of δ.

## 4. Closing Remarks

In this work, we investigated the localization properties of Aubry–André–Harper ladder networks in the presence of environmental interaction. The interaction of the system with the environment was phenomenologically incorporated by adding imaginary site energies, and depending on the sign, the interaction is referred as physical gain (positive imaginary) or physical loss (negative imaginary). We have considered four distinct configurations associated with gain/loss terms which are BT-I, IT-I, IT-II and BT-II, and for all these cases, we critically analyzed the localization phenomena. We elaborately discussed different cases of two-stranded Aubry–André–Harper ladders, and at the end, we briefly illustrated the behavior of a multi-stranded ladder as a general case. All these ladders were simulated within a tight-binding framework, which is most convenient for describing a physical system, and the localization aspects were analyzed by calculating the average inverse participation ratio. The key features and new aspects of our work are as follows:

- For the first time, to the best of our knowledge, localization phenomena were investigated considering a *multi-stranded ladder network* in the presence of environmental interaction;
- A simple prescription for decoupling any arbitrary stranded ladder system was provided. This helped us detect the mixed-phase window(s) and the mobility edge(s);
- From a comparative analysis, we established that when the gain and loss terms are added in alternate sites, maximum scattering occurs which leads to a significant impact on the localization behavior. For the ladders with either gain or loss terms, there is no effect on localization. A minor effect was noticed for the balanced type-II ladder which is very easy to understand;
- The mixed-phase window(s), and thus, the mobility edge(s) can be monitored by means of the interaction factor δ. This is an interesting observation which might be helpful in deriving controlled transport properties;
- Our analysis is not specific to any particular system—rather, we can easily extend it to any other quasicrystals in the presence of a non-Hermitian factor that exhibits a fragmented and gapped spectrum.

## Data Availability

The data that support the findings of this study are available from the corresponding author upon reasonable request.
